# Are seafloor habitats influencing the distribution of microplastics in coastal sediments of a Marine Protected Area?

**DOI:** 10.1007/s11356-023-25536-1

**Published:** 2023-02-14

**Authors:** Beatriz Rios-Fuster, Montserrat Compa, Carme Alomar, Mercè Morató, Diane Ryfer, Margarita Villalonga, Salud Deudero

**Affiliations:** Centro Oceanográfico de Baleares (IEO, CSIC), Muelle de Poniente s/n, 07015 Palma de Mallorca, Spain

**Keywords:** Anthropogenic particles, Marine Protected Area, Seafloor

## Abstract

The marine environment is affected by the increasing presence of microplastics (MPs; < 5 mm), and the seafloor acts as a sink for these particles. Locations with different predominant seafloor habitat and protection level applied were selected from Cabrera Marine-Terrestrial National Park (henceforth, Cabrera MPA) (western Mediterranean Sea) with the aim to assess the distribution of MPs along the sediments of this Mediterranean MPA. A total of 37 samples were collected. A high diversity of sediment between locations was detected according to the Udden-Wentworth classification and locations were clustered into two main groups according to the predominance of different particle size fractions. The identification of MPs was carried out according to the sediment particle size classification. A total of 1431 MPs and a mean value (± SD) of 314.53 ± 409.94 items kg^−1^ D.W. were identified, and 70% of the particles were fibers. Statistically higher abundances of MPs were found in sediments collected from sandy habitats, with a mean value of 630.80 ± 636.87 items kg^−1^ D.W., compared to the abundances of MPs found in locations with different predominant seafloor habitats, that ranged from 136.79 ± 156.33 items kg^−1^ D.W. in habitats with similar predominance of seagrass and sand to 223.02 ± 113.35 items kg^−1^ D.W. in habitats with similar predominance of rocks and sand. The abundance of MPs regarding each sediment particle size fraction differed between years and locations, and the abundance of MPs according to each identified shape differed between sampling years, particle size fraction, and predominant seafloor habitat. The present study highlights the ubiquitous presence of MPs in seafloor sediments from a MPA. Furthermore, the results suggest that the predominant seafloor habitat can modulate the presence of MPs in marine environments in both general abundances and shape of items.

## Introduction

The abundance of microplastics (MPs; < 5 mm) is continuously increasing in marine ecosystems as a consequence of their direct or indirect release into the marine environment from wastewater plants, sewage discharges (Kazour et al. 2019; Naji et al. 2021), or river effluents (Lebreton et al. 2017; Simon-Sánchez et al. 2019) amongst others. Once in the marine environment, these items can experience different fates as being ingested by biota (Compa et al. 2019; Rios-Fuster et al. 2019; Oliveira et al. 2020), colonized by different organisms creating biofilms on the surfaces of plastics (Liu et al. 2020; Wright et al. 2020), or being passively transported to other regions far from the location of their source (Compa et al. 2020b; Van Sebille et al. 2020) and throughout the water column (Dai et al. 2018).

The presence of MPs has been reported in marine habitats including the sea surface, the seafloor, and the water column (Alomar et al. 2016; Compa et al. 2020b; Rios-Fuster et al. 2022b; Fagiano et al. 2023). The physical characteristics of the MPs affect their distribution within the different marine habitats and depths of the seawater. In this sense, polymers with a higher density than seawater rapidly sink and accumulate on the seafloor (Enders et al. 2015; Eo et al. 2021). However, polymers that have a lower density than seawater are exposed to different processes, such as biofilm formation, which can alter the density of the polymer, inducing in the last instance its sinking and accumulation on the seafloor (Wu et al. 2019). In addition, the shape of the polymer can also affect the distribution of plastics within the water column since fragments can sink at a faster velocity than fibers and filaments (Kooi et al. 2016).

The Mediterranean Sea presents high concentrations of marine debris as a result of being surrounded by developed areas, but also due to the high maritime traffic routes, along with a large number of touristic and industrial activities (Grelaud & Ziveri 2020). In this sense, Marine Protected Areas (MPAs), such as the Cabrera Marine-Terrestrial National Park (henceforth, Cabrera MPA) in the western Mediterranean Sea, are indirectly exposed to this type of pollution (Blašković et al. 2017; Giovacchini et al. 2018; Compa et al. 2022b). Cabrera MPA is characterized by a rich biodiversity with endemic species such as *Posidonia oceanica* and key species such as *Pinna rudis*, *Epinephelus marginatus*, *Scorpaena scrofa*, or *Palinurus elephas*, amongst others (Reñones et al. 1997; Goñi et al. 2008; Gvozdenović et al. 2019). In addition, Cabrera MPA has an important benthic community and most of the species are filter feeders, which is a non-selective feeding behavior (Ribó et al. 2021) highlighting the importance of assessing the deposition of MPs on sediments.

It is well known that seafloor characteristics, such as sediment grain size or habitat, directly affect the distribution of marine organisms, but recently, it has been reported that it can also affect the deposition of MPs in the sediments: for example, *Posidonia oceanica* can trap and retain MPs within their structures, reducing the MP abundance from their surroundings (Sanchez-Vidal et al. 2021), and evidencing the importance to consider the predominant seafloor habitat in studies assessing the presence of MPs in sediments.

The aims of the present study are (i) to classify the sediment samples according to the predominant particle size fraction; and (ii) to detect differences in MPs accumulation based on granulometry of the sediment, the predominant habitat of the seafloor, and the protection level applied in different locations of Cabrera MPA.

## Materials and methods

### Study area

**Fig. 1:**
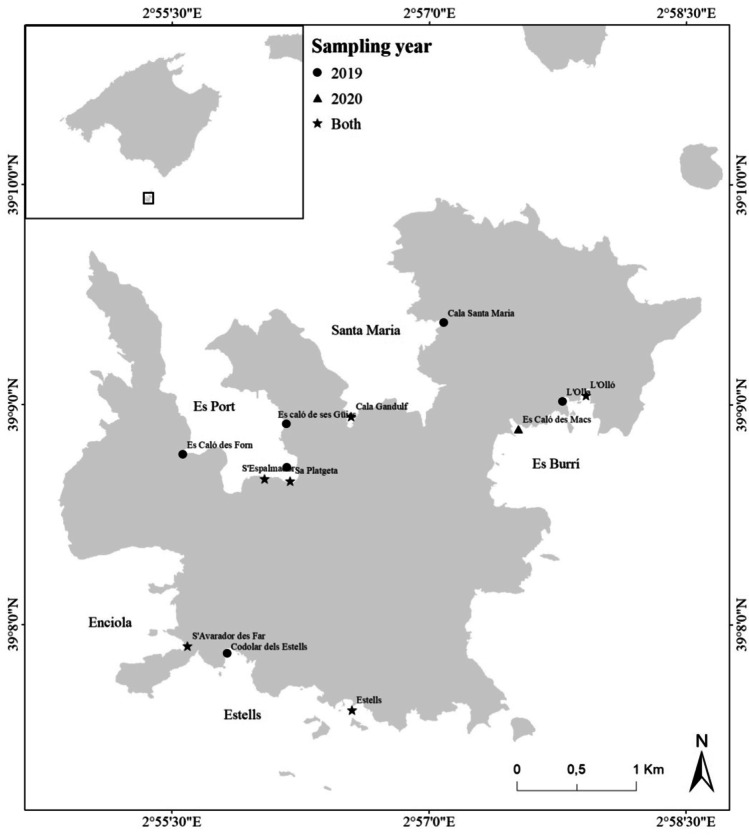
Study area with sampling locations for the quantification and identification of microplastics in coastal sediments of Cabrera National Park

The study area is located within the shallow coastal waters of the Cabrera Marine-Terrestrial National Park (henceforth, Cabrera MPA) (Fig. 1). Cabrera MPA has an area of 90,800.52 hectares, of which 89,482.52 are marine and 1318 are terrestrial, and is located on the south-eastern coast of the island of Mallorca in the Balearic Island Archipelago. The coastline has a high variety of morphologies including cliffs, coves, and bays, and is extremely rocky, with rocky outcrops on the shoreline. The samples were collected from 13 locations with different levels of protection according to the park’s legislation: sailing prohibition, and nighttime and daytime anchoring allowence in the specific and regulated Cabrera MPA buoys. The locations were classified according to the predominant seafloor habitat representing its area: rocky, sandy, seagrass, sandy and rocky, and seagrass and sandy (Table 1).

**Table 1:** Site characteristics according to protection level, predominant seafloor habitat, and mean ± standard deviation (SD) sampling depth (m)

Area	Location	MPA protection level	Predominant seafloor habitat	Mean ± SD depth (m)
Enciola	S’Avarador des Far	Sailing prohibited	Rocky	3.72 ± 3.10
Es Burrí	Es caló des Macs	Sailing prohibited	Sandy and rocky	3.93 ± 2.26
	L’Olla	Sailing prohibited	Seagrass	4.35 ± 0.88
	L’Olló	Sailing prohibited	Sandy	4.55 ± 1.81
Es Port	Es caló de ses Güies	Nighttime anchoring permitted	Seagrass	5.55 ± 3.27
	Es caló des Forn	Daytime anchoring only	Mixed	3.10 ± 0.99
	Sa Platgeta	Nighttime anchoring permitted	Sandy	1.10 ± 0.41
	Sa platgeta des Pagès	Nighttime anchoring permitted	Seagrass and sandy	2.85 ± 1.04
	S’Espalmador	Nighttime anchoring permitted	Sandy	4.29 ± 2.34
Estells	Codolar dels Estells	Sailing prohibited	Seagrass	4.10 ± 0.73
	Estells	Sailing prohibited	Rocky	4.33 ± 3.64
Santa Maria	Cala en Ganduf	Sailing prohibited	Seagrass and sandy	4.14 ± 2.15
	Cala Santa Maria	Sailing prohibited	Rocky and seagrass	3.73 ± 2.10

### Sediment samples

A total of 37 sediment samples were collected in summer months simultaneously to transects conducted in scuba diving surveys to evaluate the macrodebris along the seafloor in the study area. At each location, two sediment samples were collected. Depth was calculated as the mean depth of the initial and final depth of transects performed. During the last week of July and the first week of August 2019, two samples from 12 locations were collected, resulting in a total of 24 samples, and during the first 2 weeks of July 2020, two samples from 6 locations and an additional single sample in one location were collected, resulting in a total of 13 samples (Fig. 1). The first 5 cm of seafloor sediment was collected using a 500 ml core.

### Granulometry analysis

Once in the laboratory, 100 ml of each sediment sample was dried at 60 °C in the oven for 24–72 h depending on the moisture of each sample. Each sediment sample (ranging from 77 to 177 g depending on the sediment and grain characteristics) was placed into the sieve stack and the sample was shaken for 10 min. This sieve stack consisted of six stainless steel sieves with mesh diameters of 2, 1, 0.5, 0.25, 0.125, and 0.063 mm. The subsamples retained in each sieve and in the collector were weighed to determine the proportion of sediment in each sieve fraction. Sediment from each location was classified according to the Udden-Wentworth grain classification: 2 mm (granules), 1 mm (very coarse sand), 0.5 mm (coarse sand), 0.25 mm (fine sand), 0.125 mm (fine sand), and 0.063 mm (very fine sand) (Alomar et al. 2016). For this, the mean particle size of the sediment grains was calculated (*ϕ*) following the Udden-Wentworth scale: *ϕ* = −log_2_ (d).

### Microplastic analysis

For each of the particle size fractions, MPs were isolated and extracted following a combination of two accepted methods: density separation with saturated sodium chloride solution (NaCl) (1.2 g cm^−3^; 1:3 ratio) for samples with low organic matter content (Woodall et al. 2014), and a flotation separation process with 96% ethanol (EtOH) followed by visual sorting for vegetal-rich samples (Herrera et al. 2018). Sediment samples with NaCl were agitated for 30 s and left to settle for 5 min. The supernatant was removed and filtered through a glass fiber filter (branchia; 1.2 μm pore size and 47 mm diameter) using a vacuum pump. This process was repeated three times for each sample. For samples with a high content of organic matter, mainly with *Posidonia oceanica*, 96% EtOH was applied for flotation separation between plastic polymers and organic material. The biological material was removed and the settled fraction (containing denser plastic polymers) was dried in the oven for 24–48 h for visual sorting under the stereomicroscope and plastic identification. Direct visual sorting was done under a stereomicroscope (Euromex holland, Nexius zoom) for those subsamples with small quantity of sediment. All identified items were classified according to color and to shape as fibers, fragments, films, and ropes and filaments (Fossi et al. 2019) with 40× as maximum magnification used. Results are expressed as the mean value of the items identified in the two samples collected at each location, and as the number of items identified per kilogram of dry weight (D.W.) of sediment.

### Contamination control

At all times during laboratory work, all surfaces and instruments were thoroughly cleaned daily with EtOH and technicians wore 100% cotton laboratory coats. Throughout the process of sieving, agitation, separation, flotation, filtration, and visual sorting, blanks were placed nearby the workspace and if MPs were identified in the blanks, these were deleted from the total number of MP items found in each size fraction based on similar color and shape.

### Data analysis

A cluster dendrogram with the K-means procedure and based on Euclidean distance was performed as the ordination method for exploring differences in granulometry according to the percentages of each particle size fraction of the sediments of each location along Cabrera MPA.

A Generalized Linear Model (GLM) with a negative binomial distribution was performed to identify the main variables that affect the distribution of MPs in sediments. Three variables were introduced into the model: sampling area and predominant seafloor habitat as categorical variables and total depth as a continuous variable. This analysis has been performed to understand the variables affecting the presence of MPs along the sediments.

Additionally, to identify differences in MPs particle sizes and shapes according to environmental factors, two permutational multivariate analyses of variance (PERMANOVA) were applied. The first evaluated potential differences in the abundance of MPs from each sediment size fraction according to the sampling year, the predominant seafloor habitat, the protection level applied, sampling area, and total sampling depth. This analysis will allow us to elucidate if the factors considered play a determining role in the presence and abundance of a particular size fraction of MPs, such as whether if in the areas with the greatest traffic of people a specific particle size range of MPs is more abundant. The second PERMANOVA assessed potential differences in the abundance of MPs from each of the shapes identified according to sampling year, particle size fraction, the predominant seafloor habitat, the protection level applied, sampling area, and total sampling depth. Similarly, this analysis will allow us to elucidate if the factors considered play a determining role in the abundance of the different shapes analyzed. All data analyses were performed in RStudio version 3.6.4.

## Results and discussion

As a consequence of several physical and biological processes, the final fate of MPs is the sediments. In this study, we demonstrate the ubiquitous presence of MPs in shallow sediments from Cabrera MPA, demonstrating the high risk of exposure under which biota inhabiting the MPA are, especially those benthic species. A total of 1431 MPs with a general mean value of 314.53 ± 409.94 items kg^−1^ D.W. were found in 37 seafloor sediment samples collected from 13 locations during 2019 and 2020. The samples were mechanically shaken through sieves of 6 different mesh sizes, resulting in a total of 252 subsamples. The mean values (± SD) ranged from 64.27 ± 90.89 items kg^−1^ D.W. in Codolar dels Estells sampled in 2019 to 1248.28 ± 1451.22 items kg^−1^ D.W. in s’Espalmador sampled in 2020.

### Characterization of sediments

**Fig. 2:**
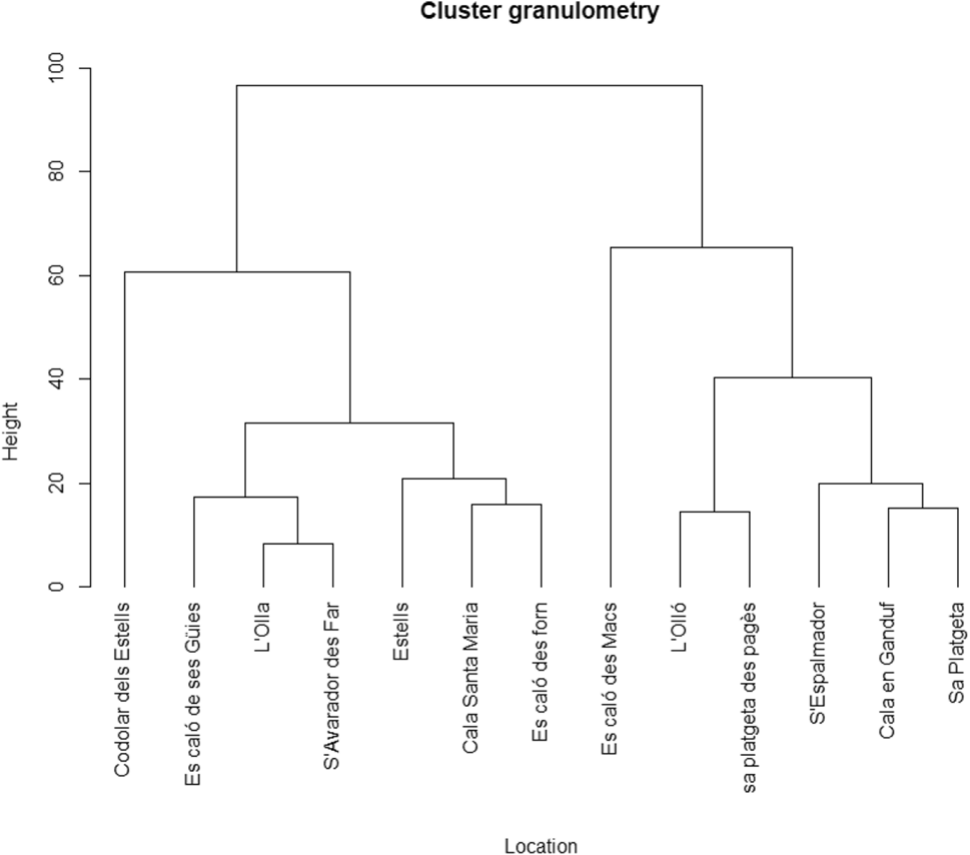
Similarities in granulometry according to the Udden-Wentworth scale among locations

Sampled locations from Cabrera MPA had different granulometry in terms of the predominant particle size fractions (Table 2; Fig. 2). The hierarchical cluster analysis showed two differentiated groups (Fig. 2). The three locations located south of Cabrera MPA, Estells, Codolar dels Estells, and s’Avarador des Far, are grouped in the same main group but show slight differences in terms of granulometry. Sediments from these three locations have a predominance for the highest particle size fractions, with 62% of the sediment in Codolar dels Estells comprised of granules (> 2 mm), while 87% of the sediment in Estells is formed by granules and coarse sand (> 2 to 0.5 mm), and 83% of the sediment of s’Avarador des Far is classified from very coarse sand to medium sand (2 to 0.25 mm). Es Caló des Forn and es Caló de ses Güies, both located in the area of Es Port, had a predominance of the highest particle size fractions (> 2 to 0.25 mm; 92% and 83% of the sediment, respectively) and were grouped jointly in the southern locations (Fig. 2). Similarly, l’Olla and Cala Santa Maria, located in different areas, were comprised of very coarse sand to medium sand (2 to 0.25 mm) (75% and 82%). Sa Platgeta, Cala en Ganduf, sa Platgeta des Pagés, and s’Espalmador did not have a clear predominance of a specific particle size fraction, and had sediments from coarse sand (1 to 0.5 mm) to very fine sand (0.125 to 0.063 mm) in percentages of 62%, 69%, 73%, and 74%, respectively. The lower fractions were predominant in Caló des Macs with the 64% of the sediments characterized as medium particle size sand fraction (0.5 to 0.25 mm) and in l’Olló with the 66% of the sediment composed by fine and very fine sand (0.25 to 0.063 mm) (Fig. 2).

**Table 2:** Summary of the mean values in percentages (%; ± SD) of the particle size fractions for each location and site. In addition, the Udden-Wentworth classification related to the particle size fraction

Area	Location	Granule	Very coarse sand	Coarse sand	Medium sand	Fine sand	Very fine sand	Silt
		> 2	2–1	1–0.5	0.5–0.25	0.25–0.125	0.125–0. 063	< 0.063
Enciola	S’Avarador des Far	15.91 ± 8.55	25.31 ± 19.50	35.47 ± 5.92	21.98 ± 23.32	0.88 ± 0.70	0.18 ± 0.08	0.28 ± 0.10
Es Burrí	Es caló des Macs	0.03 ± 0.01	0.24 ± 0.05	3.08 ± 1.48	64.44 ± 17.50	31.41 ± 18.01	0.75 ± 0.92	0.05 ± 0.00
	L’Olla	20.40 ± 28.45	20.01 ± 21.76	32.49 ± 25.20	22.91 ± 24.53	2.78 ± 1.82	1.19 ± 1.13	0.22 ± 0.21
	L’Olló	0.62 ± 0.98	2.83 ± 4.84	9.13 ± 13.52	18.93 ± 13.91	32.86 ± 13.15	33.27 ± 21.37	2.36 ± 1.53
Es Port	Es caló de ses Güies	10.50 ± 9.77	18.09 ± 9.41	27.82 ± 10.83	26.86 ± 8.14	8.89 ± 11.28	6.03 ± 8.07	1.80 ± 2.53
	Es caló des Forn	22.21 ± 9.83	31.68 ± 5.66	25.37 ± 2.23	13.24 ± 0.44	3.97 ± 2.98	2.87 ± 3.68	0.66 ± 0.18
	Sa Platgeta	13.50 ± 7.22	15.79 ± 4.60	18.93 ± 13.62	17.54 ± 5.72	25.61 ± 15.32	7.78 ± 6.06	0.85 ± 0.42
	Sa platgeta des Pagès	4.68 ± 3.30	7.21 ± 6.53	11.76 ± 9.09	22.60 ± 1.58	26.24 ± 8.29	24.24 ± 10.71	3.27 ± 2.05
	S’Espalmador	6.36 ± 2.79	12.39 ± 4.41	17.39 ± 10.80	25.62 ± 5.96	15.31 ± 11.10	16.91 ± 9.87	6.03 ± 4.00
Estells	Codolar dels Estells	61.71 ± 53.90	6.26 ± 8.80	21.73 ± 30.72	9.38 ± 13.23	0.57 ± 0.78	0.21 ± 0.23	0.14 ± 0.14
	Estells	33.18 ± 38.07	22.70 ± 15.13	31.25 ± 21.04	12.32 ± 10.77	0.26 ± 0.20	0.18 ± 0.16	0.12 ± 0.08
Santa Maria	Cala en Ganduf	11.28 ± 6.90	21.97 ± 21.23	22.15 ± 16.00	16.13 ± 11.65	14.48 ± 15.84	12.78 ± 18.76	1.22 ± 1.48
	Cala Santa Maria	17.17 ± 2.99	31.03 ± 4.06	38.62 ± 3.45	12.44 ± 2.54	0.63 ± 0.13	0.07 ± 0.02	0.04 ± 0.01

### Abundance of microplastics

#### Distribution of microplastics in Cabrera

**Fig. 3:**
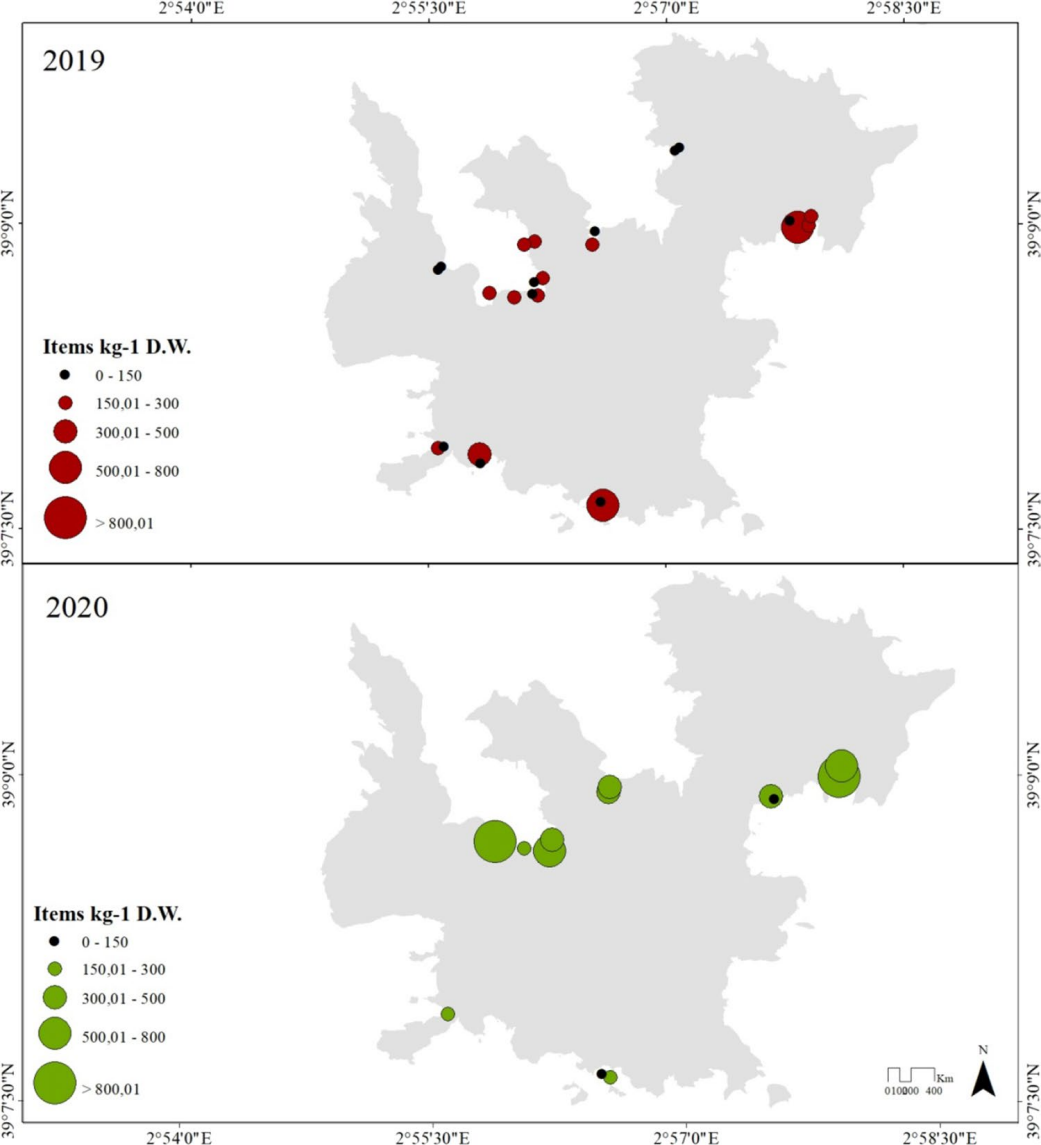
Microplastics abundances as total number of items per kilogram of dry weight of sediment (items kg^−1^) along the different sampling sites and according to sampling years

Microplastics were identified along all sampling locations showing a heterogeneous distribution (Fig. 3) but no statistically differences between sampling areas were observed (GLM, AIC = 508.97, *p* > 0.05; Table 3). Regarding the total MP items identified in sediment samples, a total of 1431 MPs with a general mean value of 314.53 ± 409.94 items kg^−1^ D.W. was found, the lower mean values of items were observed in sa platgeta des Pagès in 2019 with 8.0 ± 1.14 MPs (81.26 ± 1.57 items kg^−1^ D.W.), followed by Cala Ganduf with 9.0 ± 2.83 MPs (89.98 ± 1.35 items kg^−1^ D.W.), and es Caló des Forn with 9.0 ± 8.49 MPs (82.48 ± 82.02 items kg^−1^ D.W.) also in 2019. On the other hand, in s’Espalmador in 2020, the highest number of identified items were quantified at this location with an average of 97.78 ± 101.28 MPs (1248.28 ± 1451.22 items kg^−1^ D.W.), followed by 85.47 ± 33.28 MPs (588.53 ± 132.51 items kg^−1^ D.W.) in sa Platgeta in 2020, and 71.49 ± 21.48 MPs (800.89 ± 19.79 items kg^−1^ D.W.) found in l’Olló in 2020.

**Table 3:** Results of the generalized linear model considering sampling location, predominant seafloor habitat, and total sampling depth as coefficients. GLM, “***” *p* < 0.001, “**” *p* < 0.01, “*” *p* < 0.05, “.” *p* < 0.1. The intercept tells us the estimated value of the response variable when the continuous explanatory variables (total depth) have a value of 0, and for reference groups in categorical variables (“Enciola” regarding sampling location and “Rocky” regarding the predominant seafloor habitat)

Coefficients	Estimate	Std. error	*z* value	Pr(>|*z*|)
(Intercept)	4.96	0.62	7.96	1.77 e-15***
Area: Es Burrí	0.33	0.68	0.48	0.63
Area: Es Port	0.04	0.65	0.06	0.95
Area: Estells	0.16	0.67	0.24	0.81
Area: Santa Maria	−0.7	0.71	−0.99	0.92
Habitat: Sandy	1.03	0.51	2.02	0.04*
Habitat: Sandy and rocky	0.08	0.61	0.13	0.90
Habitat: Seagrass	−0.28	0.50	−0.57	0.57
Habitat: Seagrass and sandy	−0.39	0.56	−0.70	0.48
Depth	0.09	0.07	1.34	0.18

A previous study assessing the accumulation rate of MPs in sediments using combined radioisotope techniques detected a lower but a continuous accumulation of MPs in Cabrera MPA over time and reported values ranging from 68 to 362 MPs kg^−1^ D.W. sediment collected from Santa Maria (Dahl et al. 2021), and another study detected mean values of 0.90 ± 0.10 MPs g^−1^ D.W. and 0.24 ± 0.03 MPs g^−1^ D.W. also in Santa Maria (Alomar et al. 2016). In our study, Santa Maria showed a mean value of 86.99 ± 39.72 items kg^−1^ D.W. sediment, meaning that in the present study, the mean value of the MPs identified was intermediate-lower than the results reported by Dahl et al. (2021) and Alomar et al. (2016). Several observations can be made when comparing the findings of this study to those of other studies conducted in other worldwide regions. In samples collected from estuaries located in the Caspian coast a general mean value of 350.6 ± 232.6 MP kg^−1^ was found (Ghayebzadeh et al. 2021), similar to the general 314.53 ± 409.94 items kg^−1^ D.W. found in the present study. In Bohai bay, which is connected to the North Yellow Sea through the Bohai Strait and due to its geographical and oceanographic characteristics, it can be expected to host higher abundances of MPs than a MPA such as Cabrera National Park that showed abundances ranging from 31.1 to 256.3 MPs kg^−1^ D.W. sediment sampled (Dai et al. 2018), which are lower than those reported in the present study. Lower abundances were also detected in sediment samples collected from Andratx, a coastal urbanized and populated area with a recreational port highly exposed to anthropogenic activities located in Mallorca, and where mean values of 0.16 ± 0.09 MPs g^−1^ D.W. and 0.12 ± 0.10 MPs g^−1^ D.W. were found (Alomar et al. 2016). On the other hand, higher MP abundances in sediments were found in locations from the Spanish continental shelf with extreme values ranging from 8832 MPs kg^−1^ in Roquetas to 3819 MPs kg^−1^ in Agua Amarga, both located in the municipal area of Almería, which is known as the “plastic sea” due to the large area covered by greenhouses, which could explain these high values (Dahl et al. 2021).

Regarding the general distribution of MPs in the sediments in Cabrera MPA, no differences were observed between the sampled locations. We expected a heterogeneous distribution since the western Mediterranean Sea is affected by several currents such as the Northern Current, the Balearic Current, and the Algerian Current (Balbín et al. 2014) and our samples were collected from locations exposed to different wind, waves and currents directions and with different coastline morphologies. With this scenario, we expected a higher MP accumulation in samples collected from southern locations such as Codolar dels Estells or Estells as a consequence of a continuous transference of floating marine litter to the area due to the influence of the Algerian current (Suaria & Aliani 2014). In this sense, the Algerian basin has one of the highest abundances of floating debris in the Mediterranean Sea, as a consequence of the presence of the shipping corridor and the high maritime activity, among others (Suaria & Aliani 2014). In addition, the low abundance of MPs observed in 2019 in locations near the harbor area (es Port), such as es Caló des Forn, sa Platgeta, or sa platgeta des Pagès, is also in disagreement with the expected due to this area being one of the most crowded areas in Cabrera MPA. Despite the presence of these currents, a backtracking simulation study performed on the island of Mallorca calculated that 79% of the particles released during the exercise originated from the Balearic Islands, while only 21% were transported from other regions of the western Mediterranean such as the northern African coastline, France, and the Spanish Iberian coast (Compa et al. 2020a). The input of marine debris from the surrounding islands could explain the absence of evidence of the input of marine debris from the initially expected areas and put the focus of attention on local currents of less strength than the great currents of the western Mediterranean Sea.

#### Microplastic size fractions

**Fig. 4.**
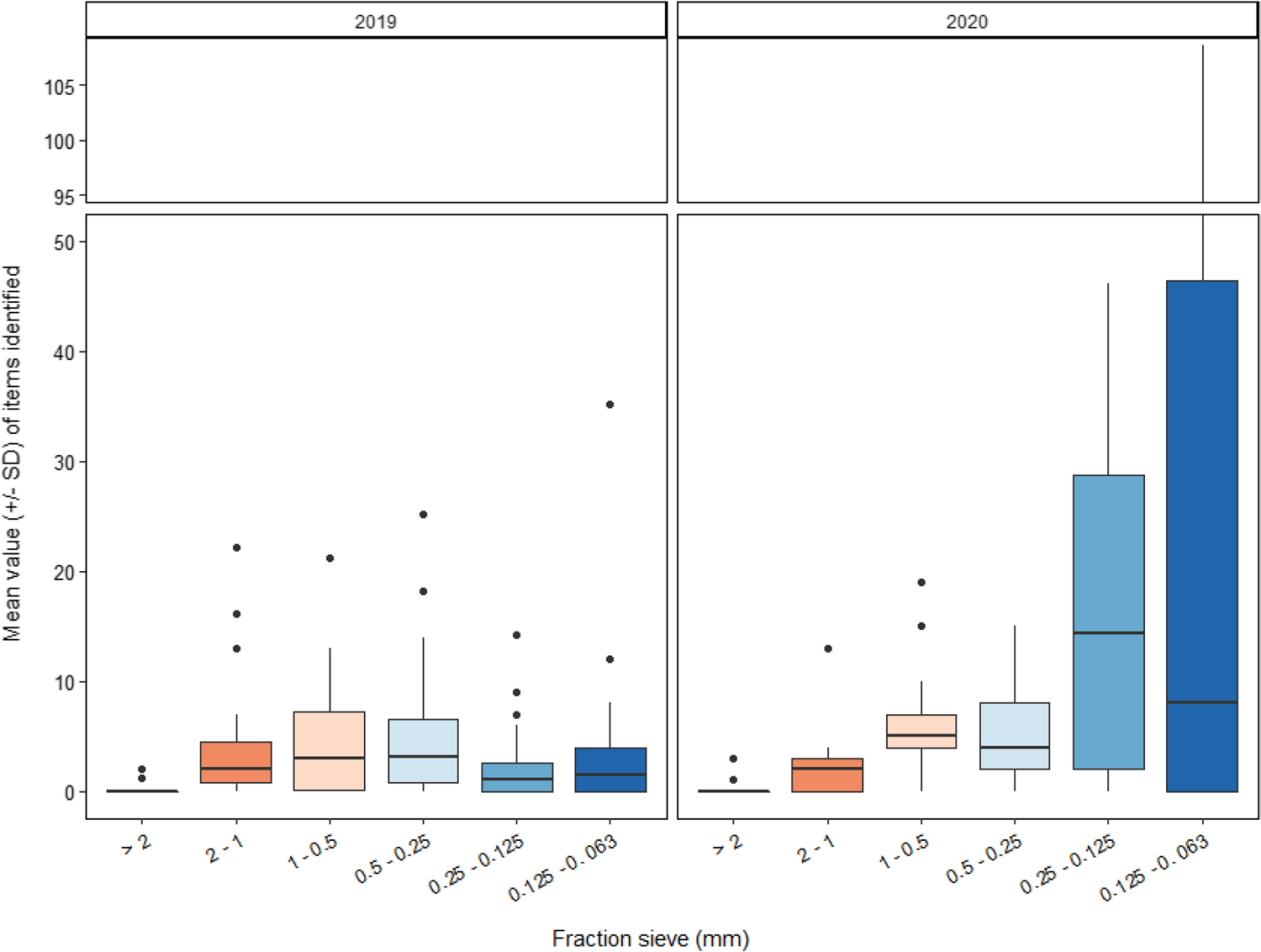
Boxplot of the mean value (±SD) of items identified at each fraction sieve according to the sampling year

The abundance of each MP size fraction showed statistical differences according to years and locations (PERMANOVA, *p* < 0.05; Table 4, Fig. 4), with an abundance of each MP size fraction from samples collected in the area of Es Port (sa Platgeta, sa Platgeta des Pagès, es caló de ses Güies, es caló des Forn, and s’Espalmador) statistically different to the abundance of each MP size fraction from samples collected at Enciola (s’Avarador des Far) (pairwise, *p* < 0.05; Table 5), but not between seafloor habitat, protection level applied, location, or total sampling depth (PERMANOVA, *p* > 0.05). In general, the abundance of MPs showed an increased trend from the highest particle size fractions to the smallest particle size fractions (Fig. 4) with the highest number of MPs identified in the smallest particle size fraction (0.125–0.063 mm) with 395 items identified and an overall mean value of 10.71 ± 21.58 items in this particle size fraction (Fig. 4), and the lowest number of MPs identified in the higher particle size fraction (> 2 cm) with 10 items identified and an overall mean value of 0.28 ± 0.77 items in this particle size fraction (Fig. 4). Although prevailing winds, waves, tides, and currents are the forces responsible for the vertical distribution and transport of MPs in sediments (Sanchez-Vidal et al. 2021; Veerasingam et al. 2021), the processes of MP deposition, retention, and resuspension in sediments are complex and still poorly understood making it difficult to comprehend the distribution of each MP size fraction. The different locations selected in the present study were characterized by different sediments in terms of granulometry, and Es Port and Enciola showed different predominant particle size fraction as observed in the cluster analysis. In this sense, locations from the area of Es Port had a predominance of the smaller particle size fraction, in comparison to other locations such as those located in the area of Estells where there is a predominance of the higher particle size fraction. In this sense, the predominance of the smaller particle size fraction may favor the rate of accumulation of MPs in sediments. The higher accumulation rate of smaller particles was previously detected in similar samples from estuarine sediments (Enders et al. 2019) and from sandy beach samples (Vermeiren et al. 2021). These results can be associated to degradation processes of plastics that are capable of generating a high number of MPs from a single macroplastic. However, an unclear trend between the particle size fraction and MP deposition was reported in a previous study conducted in the seafloor areas of Cabrera MPA, where MPs were always present in the higher particle size fractions from 0.5 to 2 mm, and when detected, highest abundances were found in the smallest size fraction (Alomar et al. 2016).

**Table 4:** Summary of the results of the permutational multivariate analysis of variance (PERMANOVA) to analyze differences in microplastic abundances in the different size fractions, and according to the shape of microplastics taking into consideration the following factors: sampling year ('Year'), particle size fraction ('Fraction'), predominant seafloor habitat ('Habitat'), protection level applied ('Protection level'), site ('Area'), and total sampling depth ('Depth'). Significant values are established at: 0 “***” 0.001 “**” 0.01 “*” 0.05 “.” 0.1 “ ” 1

Source of variation	MPs size fractions						Differences in the shape of the MPs				
	Df	SumsOfSqs	MeanSqs	F.Model	*R*^2^	Pr(>F)	SumsOfSqs	MeanSqs	F.Model	*R*^2^	Pr(>F)
Year	1	0.5914	0.5914	2.7094	0.069	0.01**	4.937	4.9374	22.9965	0.1265	0.001***
Fraction	5	--	--	--	--	--	3.448	0.6896	3.2118	0.0883	0.001***
Habitat	4	0.8947	0.2238	1.0247	0.104	0.46	2.178	0.5444	2.5358	0.0558	0.002**
Protection level	2	0.3007	0.1503	0.6887	0.035	0.80	0.608	0.3039	1.4153	0.0156	0.179
Area	3	1.1813	0.3938	1.0804	0.138	0.03*	3.172	0.2883	1.3429	0.0813	0.075 .
Depth	1	0.1616	0.1616	0.7405	0.019	0.65	0.426	0.4262	1.9850	0.0109	0.103
Residuals	25	5.4572	0.2183		0.636		24.262	0.2147		0.6216	
Total	36	8.5868			1.0		39.030			1.0	

Signif. codes: 0 “***” 0.001 “**” 0.01 “*” 0.05 “.” 0.1 “ ” 1

**Table 5:** Pairwise comparisons using permutation MANOVAs on a distance matrix to analyze differences between the total set of abundances of the different MP size fractions identified at the different locations to analyze differences between the total set of the shapes of the microplastics identified at the different particle size fraction, and to analyze differences between the total set of the shapes of the microplastics identified at the different seafloor habitats. Significant differences are highlighted in bold

Pairwise regarding total set of abundances of the different MP size fractions						
			Enciola	Es Burrí	Es Port	Estells
Es Burrí			0.056	-	-	-
Es Port			**0.011**	0.686	-	-
Estells			1.000	0.153	0.057	-
Santa Maria			0.357	1.000	1.000	1.000
Pairwise regarding total set of shapes of the items identified at each of the particle size fractions studied						
	> 2	2–1	1–0.5	0.5–0.25	0.25–0.125	0.125–0.063
0.125–0.063	0.155	-	-	-	-	-
0.25–0.125	0.058	0.950	-	-	-	-
0.5–0.25	0.175	**0.015**	0.082	-	-	-
1–0.5	0.428	0.137	0.181	1.000	-	-
2–1	0.863	0.051	**0.042**	1.000	1.000	-
Pairwise regarding total set of shapes of the items identified at the different habitats						
			Rocky	Sandy	Sandy and rocky	Seagrass
Sandy			0.2532		-	-
Sandy and rocky			1.000	1.000	-	-
Seagrass			**0.0045**	**0.0020**	**0.0045**	-
Seagrass and sandy			1.000	1.000	1.000	**0.0196**

Furthermore, with respect to the shape of MPs identified with sizes ranging from 0.5 to 0.25 mm, they were statistically different compared to the shapes of MPs with sizes ranging from 0.125 to 0. 063 mm (pairwise, *p* < 0.05; Table 5). The percentages of fibers were equal or higher to 50% of the items identified in all the particle size fractions, lower than in the very coarse sand (2 to 1 mm); were fibers made up 25% of the total items identified (Fig. 7a). Differences regarding the abundance of each type of shape identified regarding the MP size fraction suggest an unequal degradation process of the different polymers depending on their shapes. For example, Styrofoam particles were only identified with sizes ranging from 2 to 1 mm, and filaments were only observed in sizes ranging from 0.5 to 0.25 mm. This result highlights the ubiquity of small fibers (ranging from 0.25 to 0.065 mm) within the sediments from Cabrera MPA. The ubiquity of fibers has previously been reported at different depths of the water column along the Spanish Mediterranean coast in the western Mediterranean Sea, and although higher abundances were reported near the sea surface (at 5 m depth) and showed a decrease in the water column, the presence of fibers was observed at all sampling depths studied (Rios-Fuster et al. 2022b).

#### Differences between sampling years

The abundances of MPs differed between the two sampling years with higher values in 2020. In 2019, higher abundances of MPs with sizes ranging from 1 to 0.25 mm were observed, while in 2020, the MP abundances gradually increased from the higher particle size fraction (> 2 mm) to the residual particle size fraction (< 0.063 mm) (Fig. 4). Regarding the shapes of the items identified, 70% of the MPs collected were fibers and the second most common items were fragments (21%) followed by films (3.7%). Of these items, in 2019, 89% of the items were fibers while in 2020, fibers decreased to 57% of the total amount of identified items while fragments were the second most common shape comprising 30% of the identified items. Locations from the harbor area (es Port) showed higher abundances of MPs in 2020 than in 2019. The differences between years suggest that some meteorological event could have occurred that caused a greater input of MPs to the coasts of Cabrera, and that due to the characteristics of the coast, with several enclosed bays with low hydrodynamics, MPs got trapped in the surrounding waters. Additionally, it should be taken into account that the second sampling survey was carried out during the pandemic restrictions (COVID-19) and that the parks' cleaning tasks were affected causing a greater accumulation of macroplastics. In this sense, the effective removal of macro-marine debris would reduce the generation of MPs sunken into the sediment since it will avoid the degradation of macroplastics into MPs. In addition, mitigation measures are more effective with macro-marine debris rather than with micro-marine debris, as larger items are easier to detect and remove than microparticles. However, comparing 2 years of data has several gaps such as the difficulty to correlate any difference observed with external factors. For example, it is not possible to clarify if a higher abundance is a consequence of a punctual event, and a long-term monitoring is advisable in order to detect temporal fluctuations and to understand the factors affecting to them.

### External factors under study

#### Influence of the predominant seafloor habitat

**Fig. 5:**
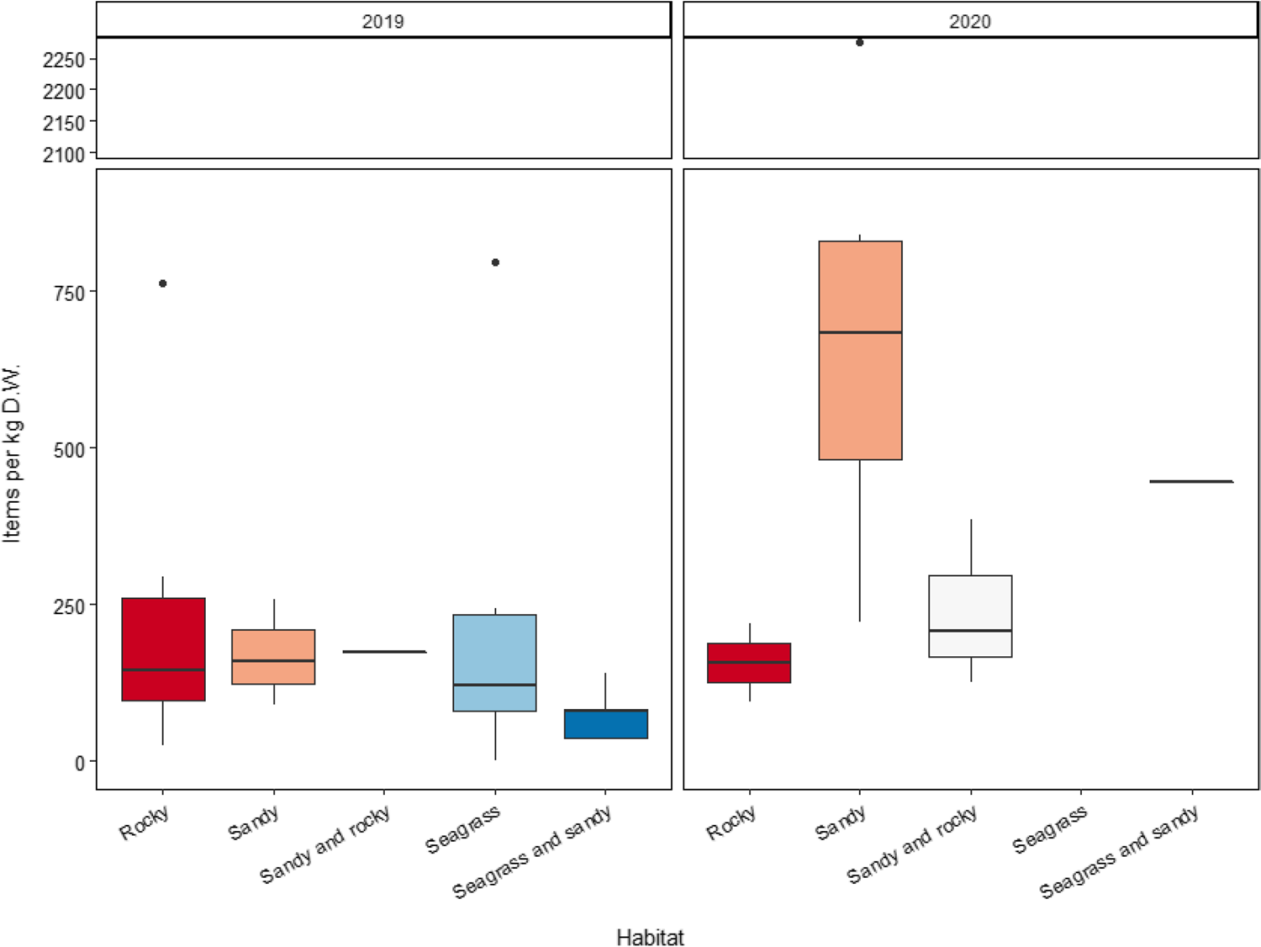
Boxplot of the items per kilogram (items kg^−1^ D.W.) identified at each habitat according to the sampling year

The abundance of MPs at the different locations was statistically different between habitats (GLM, AIC = 506.88, *p* < 0.05; Table 3). A heterogenic abundance of MPs according to the predominant seafloor habitat was observed being the sandy habitat where the highest abundances of MPs were found with a total of 610 identified items and a mean value of 630.80 ± 636.87 items kg^−1^ D.W., followed by samples collected in rocky habitats with a total of 237 identified items and a mean value of 218.10 ± 221.14 items kg^−1^ D.W. (Fig. 5). In general, the lower values were located in samples were seagrass and sand predominate with 87 items identified and a mean value of 136.79 ± 156.33 items kg^−1^ D.W. (Fig. 5). A heterogenic abundance of MPs was observed according to the predominant seafloor habitat being the sandy habitat from samples collected in 2020 where the highest abundances of MPs were found with a total of 610 items identified and a mean value of 630.80 ± 636.87 items kg^−1^ D.W., followed by samples collected in seafloor habitats in which predominates both seagrass and sand with a mean value of 444.64 ± NA items kg^−1^ D.W. (Fig. 5). In general, the lower values were located in 2019 in the seagrass and sandy predominant seafloor habitat and the rocky predominant seafloor habitat with mean values ranging from 34.31 ± NA to 292.69 ± 325.56 items kg^−1^ D.W., respectively (Fig. 5). No samples from locations were the predominant seafloor habitat was composed by seagrass were collected in 2020 (Fig. 5).

The shape of the MP was statistically different between years, particle size fraction, and predominant seafloor habitat (PERMANOVA, *p* < 0.05; Table 4), but not between sampling locations and total depth (PERMANOVA, *p* > 0.05). The mixed habitat of seagrass and sand had the lowest percentage of fibers, with only 30% of fibers and approximately 70% of fragments (Fig. 7b). In seagrass and rocky seafloor habitats, 99% and 90% of the items, respectively, were fibers (Fig. 7b). The shape of the items identified in the seagrass habitats was statistically different from the shape of the items identified in the other habitats (pairwise *p* < 0.05; Table 5), where more heterogenic shapes were identified. Furthermore, seafloor habitats composed of a mixture of seagrass and sand have the highest percentage of fragments, and seafloor habitats composed of a mixture of sand and rocks have the highest percentage of films compared to the other habitats. This fact can be explained by the phenomenon mentioned above that exposes the ability of some vegetal species such as *Posidonia oceanica* to trap MPs generating aegagropilaes jointly with the lignocellulosic debris of *P. oceanica* which under extreme meteorological conditions are washed ashore, reducing the presence of these particles in the water environment (Sanchez-Vidal et al. 2021). In addition to the presence of relevant species such as *P. oceanica*, some benthic species inhabiting the area such as sea cucumbers can alter the distribution of MPs in marine sediments as a consequence of the high ingestion of MPs and the consequent accumulation of these particles due to its non-selective feeding behavior (Compa et al. 2022a; Rios-Fuster et al. 2022a).

In our study, sandy seafloors have been detected to accumulate the highest abundances of MPs in Cabrera MPA in contradistinction to the study performed in the Florida Keys where a higher MP mean abundance in seagrass beds was detected in comparison to the adjacent sand flats (Plee & Pomory 2020). Regarding the role of the seafloor environment in the fate of MPs in sediments reported in other studies, specific habitat characteristics have been reported to promote MP accumulation in sediments (Huang et al. 2020; Esiukova et al. 2021; Sanchez-Vidal et al. 2021). A previous study detected that MP abundances are higher within a factor of 2.9 in seagrass meadows than in non-vegetated areas (Huang et al. 2020), possibly due to the reduction of the flow as a consequence of the presence of the vegetation that can generate areas of accumulation of marine debris (Sanchez-Vidal et al. 2021). In addition, other study reported that dry algae mass samples showed MPs abundances with one order of magnitude higher than sand samples (Esiukova et al. 2021). All these studies suggest that seagrass meadows and algae have the ability to trap MPs with the potential effects to the general functioning of the related ecosystems (Bonanno & Orlando-Bonaca 2020) like the reported physical, physiological, and genetical effects in primary producers such as microalgae (Lagarde et al. 2016; Zhang et al. 2017; Wang et al. 2019).

The presence of MPs in the different habitats can affect organisms associated to the seafloor as MPs located within the sediment became easier available for epibenthic species, such as the lugworm *Arenicola marina* in which the ingestion of MP has already been reported (Besseling et al. 2013); meanwhile, MPs present at deeper layers of sediments became more available to species inhabiting the sediment such as the bivalve *Scrobicularia plana* or the ragworm *Hediste diversicolor* in which the ingestion of MP has also already been reported (Ribeiro et al. 2017; Silva et al. 2020). In this sense, MPs can affect differently according to their physical characteristics, and survival rates of the adult daggerblade grass shrimp (*Palaemonetes pugio*) towards MPs ingestion has been detected to be affected by the size and the shape of the MPs (Gray & Weinstein 2017). Different zooplankton species showed a MP shape preference and this was explained by the different feeding behaviors performed by these species since *Calanus helgolandicus* as a suspension feeder ingested significantly more fragments than fibers and beads, *Acartia tonsa* an ambush feeder ingested significantly more fibers, and *Homarus gammarus* larvae, also an ambush feeder, more beads (Botterell et al. 2020). In addition, all individuals analyzed from Cabrera MPA of *Holothuria tubulosa*, *Holothuria poli*, and *Holothuria forskali* showed a high MP ingestion with the 100% of the individuals showing MPs, mainly fibers, in their gastrointestinal tract (Rios-Fuster et al. 2022a).

#### The effect of the protection level applied

**Fig. 6:**
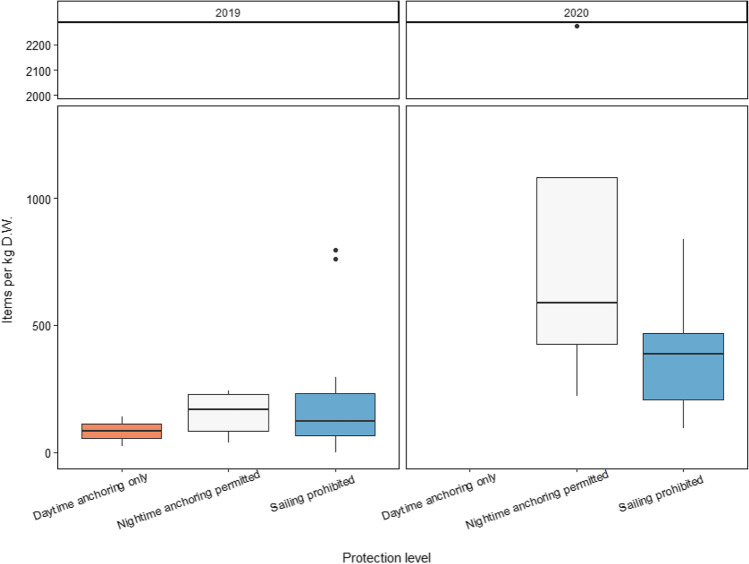
Boxplot of the items per kilogram (items kg^−1^ D.W.) identified at each protection level according to the sampling year

Different levels of protection did not affect the abundances of MPs in Cabrera MPA as no statistical differences were observed between sampling locations with respect to the restrictions applied (Table 4; Fig. 6). There was an increase in MP abundances from locations in which the nighttime anchoring is allowed in 2020 with a mean value of 918.40 ± 923.56 items kg^−1^ D.W. A previous study assessing marine debris distribution in Cabrera MPA with underwater scuba diving surveys reported no differences of macro-marine debris abundances between areas with different protection status neither for the average number of marine debris nor for the weight of marine debris (Compa et al. 2022b). As sediments represent a potential final fate of MPs in the marine environment, MPs found in sediments from Cabrera MPA could be the result of continuous input from other areas. Our results confirm the transference of MPs from urban to protected areas most probably as a consequence of oceanographic currents. Additionally, we can suggest that the restrictions applied are not enough to avoid the accumulation of MPs in sediments from a MPA since the accumulation of these particles is ubiquity along the shallow sediments with different protection levels applied, and reinforce the idea of the requirement to toughen the mitigation measures.

**Fig. 7:**
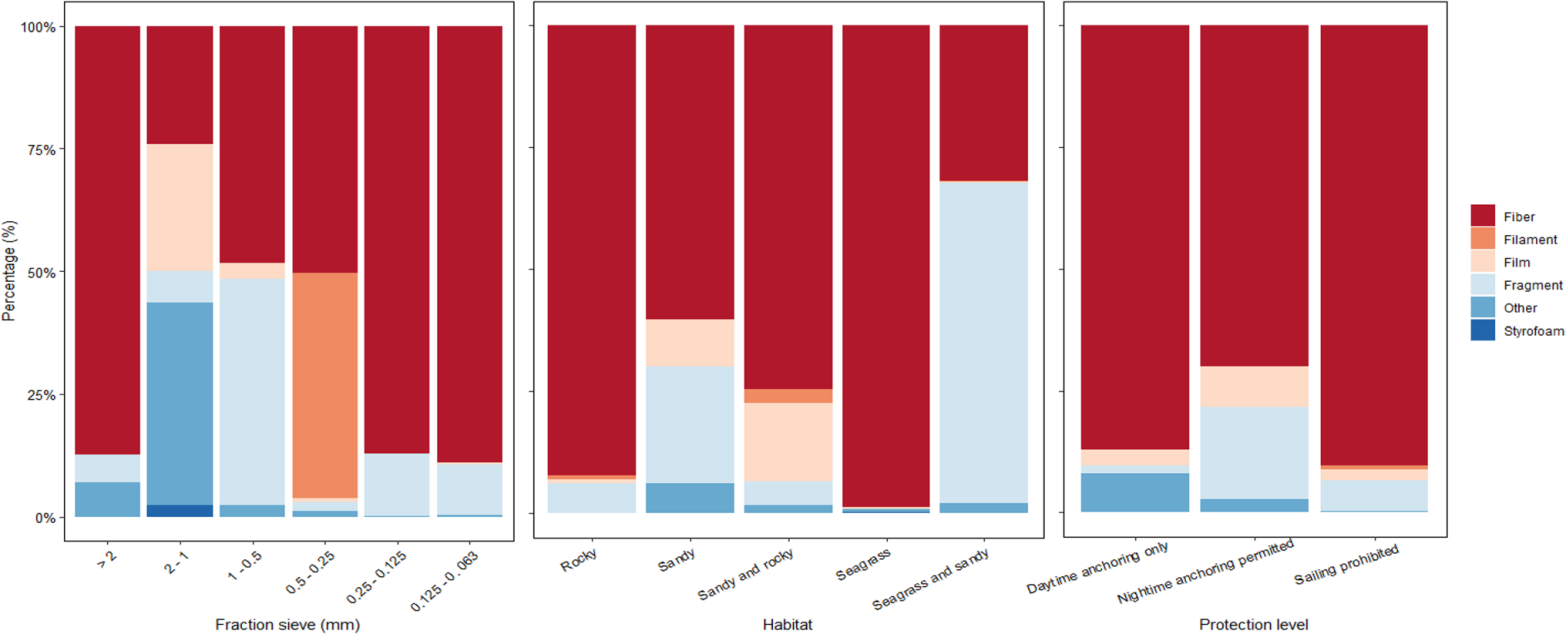
Stacked barplot representing the percentage of each microplastic shape (fiber, filament, film, fragment, Styrofoam, and other) identified according to each sediment fraction, habitat, and protection level

Regarding the shape of the items identified, more than 85% of the items identified in locations in which only daytime anchoring is permitted and also in which sailing is prohibited were fibers (Fig. 7c). In locations in which the nighttime anchoring is allowed, the percentage of fibers was approximately 70% of the total items identified, followed by a 20% of fragments (Fig. 7c). As previously suggested, the higher anthropogenic impact performed in those areas where the nighttime anchoring is allowed can be affecting the general input of fragments to the environment, suggesting the requirement of stronger mitigation measures in the park.

## Conclusions

The present study demonstrates the ubiquity of MPs in shallow sediments from the Archipelago of Cabrera, a MPA from the western Mediterranean Sea. Cabrera MPA has a heterogenic diversity in terms of sediment granulometry that can be altering the deposition rate of MPs in seafloor areas. In addition, results from the present study highlight that the diversity of the seafloor is able to modulate the presence of MPs in the marine environments in both, general abundances, being sandy habitats those in which higher abundances of MPs are found, and in the shape of the items present, being habitats with predominance of seagrass those in which almost the totality of the items identified are fibers, in contraposition to the other habitat types in which different shapes are identified. Additionally, results suggest that the shapes of the MPs are represented differently according to particle size fraction, being fibers with sizes within the smaller size fraction detected in a higher proportion, and the other shapes such as fragments, filaments, and films, only being present in the intermediate sizes range.

## References

[CR1] Alomar C, Estarellas F, Deudero S (2016). Microplastics in the Mediterranean Sea: deposition in coastal shallow sediments, spatial variation and preferential grain size. Mar Environ Res.

[CR2] Balbín R, López-Jurado JL, Flexas MM, Reglero P, Vélez-Velchí P, González-Pola C, Rodríguez JM, García A, Alemany F (2014). Interannual variability of the early summer circulation around the Balearic Islands: driving factors and potential effects on the marine ecosystem. J Mar Syst.

[CR3] Besseling E, Wegner A, Foekema EM, Van Den Heuvel-Greve MJ, Koelmans AA (2013). Effects of microplastic on fitness and PCB bioaccumulation by the lugworm Arenicola marina (L.). Environ Sci Technol.

[CR4] Blašković A, Fastelli P, Čižmek H, Guerranti C, Renzi M (2017). Plastic litter in sediments from the Croatian marine protected area of the natural park of TelašČica bay (Adriatic Sea). Mar Pollut Bull.

[CR5] Bonanno G, Orlando-Bonaca M (2020). Marine plastics: what risks and policies exist for seagrass ecosystems in the Plasticene?. Mar Pollut Bull.

[CR6] Botterell ZLR, Beaumont N, Cole M, Hopkins FE, Steinke M, Thompson RC, Lindeque PK. 2020. Bioavailability of microplastics to marine zooplankton: effect of shape and infochemicals. 10.1021/acs.est.0c02715 32927944

[CR7] Compa M, Alomar C, Mourre B, March D, Tintor J, Deudero S. 2020a. Nearshore spatio-temporal sea surface trawls of plastic debris in the Balearic Islands 158. 10.1016/j.marenvres.2020.104945 32217295

[CR8] Compa M, Alomar C, Mourre B, March D, Tintoré J, Deudero S (2020b) Nearshore spatio-temporal sea surface trawls of plastic debris in the Balearic Islands. Mar Environ Res 158 10.1016/j.marenvres.2020.104945 32217295

[CR9] Compa M, Alomar C, López Cortès MF, Rios-fuster B, Morató M, Capó X, Fagiano V, Deudero S. 2022a. Multispecies assessment of anthropogenic particle ingestion in a Marine Protected Area. biology. 10.3390/biology11101375 PMC9598462 36290281

[CR10] Compa M, Alomar C, Wilcox C, van Sebille E, Lebreton L, Hardesty BD, Deudero S (2019). Risk assessment of plastic pollution on marine diversity in the Mediterranean Sea. Sci Total Environ.

[CR11] Compa M, Alomar C, Morató M, Álvarez E, Deudero S (2022). Are the seafloors of marine protected areas sinks for marine litter? Composition and spatial distribution in Cabrera National Park. Sci Total Environ.

[CR12] Dahl M et al (2021) A temporal record of microplastic pollution in Mediterranean seagrass soils. Environ Pollut 273 10.1016/j.envpol.2021.116451 33486243

[CR13] Dai Z, Zhang H, Zhou Q, Tian Y, Chen T, Tu C, Fu C, Luo Y (2018). Occurrence of microplastics in the water column and sediment in an inland sea affected by intensive anthropogenic activities. Environ Pollut.

[CR14] Enders K (2019). Tracing microplastics in aquatic environments based on sediment analogies. Sci Rep.

[CR15] Enders K, Lenz R, Stedmon CA, Nielsen TG (2015). Abundance, size and polymer composition of marine microplastics ≥10 μm in the Atlantic Ocean and their modelled vertical distribution. Mar Pollut Bull.

[CR16] Eo S, Hong SH, Song YK, Han GM, Seo S, Shim WJ (2021). Prevalence of small high-density microplastics in the continental shelf and deep sea waters of East Asia. Water Res.

[CR17] Esiukova EE, Lobchuk OI, Volodina AA, Chubarenko IP (2021). Marine macrophytes retain microplastics. Mar Pollut Bull.

[CR18] Fagiano V, Compa M, Alomar C, Rios-Fuster B, Morató M, Capó X, Deudero S (2023). Breaking the paradigm: marine sediments hold two-fold microplastics than sea surface waters and are dominated by fibers. Sci Total Environ.

[CR19] Fossi M. et al. 2019. Toolkit for monitoring ML and its impacts on biodiversity in Med MPAs.

[CR20] Ghayebzadeh M, Taghipour H, Aslani H (2021). Abundance and distribution of microplastics in the sediments of the estuary of seventeen rivers: Caspian southern coasts. Mar Pollut Bull.

[CR21] Giovacchini A, Merlino S, Locritani M, Stroobant M (2018). Spatial distribution of marine litter along italian coastal areas in the Pelagos sanctuary (Ligurian Sea-NW Mediterranean Sea): a focus on natural and urban beaches. Mar Pollut Bull.

[CR22] Goñi R (2008). Spillover from six western Mediterranean marine protected areas: evidence from artisanal fisheries. Mar Ecol Prog Ser.

[CR23] Gray AD, Weinstein JE. 2017. Size and shape dependent effects of microplastic particles on adult daggerblade grass shrimp, Palaemonetes pugio. Environmental Toxicology. 10.1002/etc.3881 28594093

[CR24] Grelaud M, Ziveri P (2020). The generation of marine litter in Mediterranean island beaches as an effect of tourism and its mitigation. Sci Rep.

[CR25] Gvozdenović S, Mačić V, Pešić V, Nikolić M, Peraš I, Mandić M (2019). Review on pinna rudis (Linnaeus, 1758) (bivalvia: Pinnidae) presence in the Mediterranean. Agric Forestry.

[CR26] Herrera A, Garrido-Amador P, Martínez I, Samper MD, López-Martínez J, Gómez M, Packard TT (2018). Novel methodology to isolate microplastics from vegetal-rich samples. Mar Pollut Bull.

[CR27] Huang Y, Xiao X, Xu C, Perianen YD, Hu J, Holmer M (2020). Seagrass beds acting as a trap of microplastics — emerging hotspot in the coastal region?. Environ Pollut.

[CR28] Kazour M, Terki S, Rabhi K, Jemaa S, Khalaf G, Amara R (2019). Sources of microplastics pollution in the marine environment: importance of wastewater treatment plant and coastal landfill. Mar Pollut Bull.

[CR29] Kooi M et al. 2016. The effect of particle properties on the depth profile of buoyant plastics in the ocean. Nature Publishing Group:1–10. Nature Publishing Group. 10.1038/srep33882 PMC5056413 27721460

[CR30] Lebreton LCM, Van Der Zwet J, Damsteeg JW, Slat B, Andrady A, Reisser J (2017). River plastic emissions to the world’s oceans. Nat Commun.

[CR31] Liu P, Zhan X, Wu X, Li J, Wang H, Gao S (2020) Effect of weathering on environmental behavior of microplastics: properties, sorption and potential risks. Chemosphere 242 Elsevier B.V 10.1016/j.chemosphere.2019.125193 31678851

[CR32] Naji A, Azadkhah S, Farahani H, Uddin S, Khan FR (2021). Microplastics in wastewater outlets of Bandar Abbas city (Iran): a potential point source of microplastics into the Persian Gulf. Chemosphere.

[CR33] Oliveira AR, Sardinha-Silva A, Andrews PLR, Green D, Cooke GM, Hall S, Blackburn K, Sykes AV (2020) Microplastics presence in cultured and wild-caught cuttlefish, Sepia officinalis. Mar Pollut Bull 160 10.1016/j.marpolbul.2020.111553 32810671

[CR34] Plee TA, Pomory CM (2020). Microplastics in sandy environments in the Florida Keys and the panhandle of Florida, and the ingestion by sea cucumbers (Echinodermata: Holothuroidea) and sand dollars (Echinodermata: Echinoidea). Mar Pollut Bull.

[CR35] Reñones O, Moranta J, Coll J, Morales-Nin B (1997). Rocky bottom fish communities of Cabrera Archipelago National Park (Mallorca, Western Mediterranean). Sci Mar.

[CR36] Ribeiro F, Garcia AR, Pereira BP, Fonseca M, Mestre NC, Fonseca TG, Ilharco LM, Bebianno MJ (2017). Microplastics effects in Scrobicularia plana. Mar Pollut Bull.

[CR37] Ribó M, Macdonald H, Watson SJ, Hillman JR, Strachan LJ, Thrush SF, Mountjoy JJ, Hadfield MG, Lamarche G (2021) Predicting habitat suitability of filter-feeder communities in a shallow marine environment, New Zealand. Mar Environ Res 163 10.1016/j.marenvres.2020.105218 33385975

[CR38] Rios-Fuster B, Alomar C, Compa M, Guijarro B, Deudero S (2019). Anthropogenic particles ingestion in fish species from two areas of the western Mediterranean Sea. Mar Pollut Bull.

[CR39] Rios-Fuster B, Alomar C, Paniagua González G, Soliz Rojas DL, Fernández Hernando P, Garcinuño Martínez RM, Deudero S (2022a) Assessing microplastic ingestion and occurrence of bisphenols and phthalates in bivalves, fish and holothurians from a Mediterranean Marine Protected Area. Environ Res 214 10.1016/j.envres.2022.114034 35948144

[CR40] Rios-Fuster B, Compa M, Alomar C, Fagiano V, Ventero A, Iglesias M, Deudero S (2022). Ubiquitous vertical distribution of microfibers within the upper epipelagic layer of the western Mediterranean Sea. Estuar Coast Shelf Sci.

[CR41] Sanchez-Vidal A, Canals M, de Haan WP, Romero J, Veny M (2021). Seagrasses provide a novel ecosystem service by trapping marine plastics. Scientific Reports.

[CR42] Silva MSS, Oliveira M, Valente P, Figueira E, Martins M, Pires A (2020). Behavior and biochemical responses of the polychaeta Hediste diversicolor to polystyrene nanoplastics. Sci Total Environ.

[CR43] Simon-Sánchez L, Grelaud M, Garcia-Orellana J, Ziveri P (2019). River Deltas as hotspots of microplastic accumulation: the case study of the Ebro River (NW Mediterranean). Sci Total Environ.

[CR44] Suaria G, Aliani S (2014). Floating debris in the Mediterranean Sea. Mar Pollut Bull.

[CR45] Van Sebille E et al. 2020. The physical oceanography of the transport of floating marine debris. Environmental Research Letters 15. IOP Publishing.

[CR46] Veerasingam S, Vethamony P, Aboobacker VM, Giraldes AE, Dib S, Al-Khayat JA (2021). Factors influencing the vertical distribution of microplastics in the beach sediments around the Ras Rakan Island, Qatar. Environ Sci Pollut Res.

[CR47] Vermeiren P, Lercari D, Muñoz CC, Ikejima K, Celentano E, Jorge-Romero G, Defeo O (2021) Sediment grain size determines microplastic exposure landscapes for sandy beach macroinfauna. Environ Pollut 286 10.1016/j.envpol.2021.117308 33991734

[CR48] Wang W, Gao H, Jin S, Li R, Na G (2019). The ecotoxicological effects of microplastics on aquatic food web, from primary producer to human: a review. Ecotoxicol Environ Saf.

[CR49] Woodall LC, Sanchez-vidal A, Paterson GLJ, Coppock R, Sleight V, Calafat A, Rogers AD, Narayanaswamy BE, Thompson RC (2014). The deepsea major sink for microplastic. R Soc Open Sci.

[CR50] Wright RJ, Erni-Cassola G, Zadjelovic V, Latva M, Christie-Oleza JA (2020). Marine plastic debris: a new surface for microbial colonization. Environ Sci Technol.

[CR51] Wu P (2019). Environmental occurrences, fate, and impacts of microplastics. Ecotoxicol Environ Saf.

[CR52] Zhang C, Chen X, Wang J, Tan L (2017). Toxic effects of microplastic on marine microalgae Skeletonema costatum: interactions between microplastic and algae. Environ Pollut.

[CR53] Lagarde F, Olivier O, Zanella M, Daniel P, Hiard S, Caruso A (2016). Microplastic interactions with freshwater microalgae: hetero-aggregation and changes in plastic density appear strongly dependent on polymer type. Environ Pollut.

